# 3-(2-Amino­eth­yl)-2-[4-(trifluoro­meth­oxy)anilino]quinazolin-4(3*H*)-one

**DOI:** 10.1107/S1600536810024645

**Published:** 2010-06-30

**Authors:** Zhao-Hui Cai, Xiao-Bao Chen, Xu-Hong Yang, Xiang Wang

**Affiliations:** a Department of Chemistry and Life Science, Xianning College, Xianning 4371000, Hubei, People’s Republic of China; b Department of Medicinal Chemistry, Yunyang Medical College, Shiyan 442000, Hubei, People’s Republic of China; c College of Life and Environmental Science, Kaili University, Kaili 556000, Guizhou, People’s Republic of China

## Abstract

In the title compound, C_17_H_15_F_3_N_4_O_2_, the dihedral angle between the trifluoro­meth­oxy-substituted benzene ring and the pyrimidinone ring is 45.1 (5)°, while that between the fused benzene ring and the pyrimidinone ring is 0.67 (1)°. Part of one of the benzene rings and its trifluoro­meth­oxy substituent are disordered over two positions of approximately equal occupancy (0.51:0.49). Inter­molecular N—H⋯O and N—H⋯N hydrogen bonds contribute to the stability of the crystal structure. A weak intra­molecular C—H⋯F contact is also found. In addition, π–π stacking inter­actions, with centroid–centroid distances in the range 3.673 (6)–3.780 (8) Å, and weak C—H⋯π inter­actions are also observed.

## Related literature

For the biological activity of quinazoline-4(3*H*)-one derivatives, see: Pandeya *et al.*(1999 ▶); Shiba *et al.* (1997 ▶), Malamas & Millen (1991 ▶); Mannschreck *et al.* (1984 ▶); Kung *et al.* (1999 ▶); Bartroli *et al.* (1998 ▶); Palmer *et al.* (1997 ▶); Tsou *et al.* (2001 ▶); Matsuno *et al.* (2002 ▶). For the synthesis of the title compound, see: Yang *et al.* (2008 ▶).
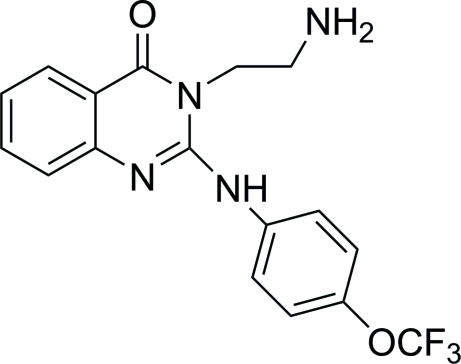


## Experimental

### 

#### Crystal data

C_17_H_15_F_3_N_4_O_2_
*M* *_r_* = 364.33
Orthorhombic, 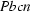

*a* = 11.9675 (13) Å
*b* = 12.9579 (13) Å
*c* = 21.280 (2) Å
*V* = 3300.0 (6) Å^3^
*Z* = 8
Mo *K*α radiation
μ = 0.12 mm^−1^
*T* = 298 K
0.23 × 0.15 × 0.11 mm

#### Data collection

Bruker SMART APEX CCD area-detector diffractometer
Absorption correction: multi-scan (*SADABS*; Sheldrick, 2001 ▶) *T*_min_ = 0.973, *T*_max_ = 0.987
15599 measured reflections
3076 independent reflections
2573 reflections with *I* > 2σ(*I*)
*R*_int_ = 0.083

#### Refinement

*R*[*F* ^2^ > 2σ(*F* ^2^)] = 0.061
*wR*(*F* ^2^) = 0.164
*S* = 1.12
3076 reflections
311 parameters
19 restraints
H atoms treated by a mixture of independent and constrained refinement
Δρ_max_ = 0.39 e Å^−3^
Δρ_min_ = −0.29 e Å^−3^

### 

Data collection: *SMART* (Bruker, 2000 ▶); cell refinement: *SAINT* (Bruker, 2000 ▶); data reduction: *SAINT*; program(s) used to solve structure: *SHELXS97* (Sheldrick, 2008 ▶); program(s) used to refine structure: *SHELXL97* (Sheldrick, 2008 ▶); molecular graphics: *SHELXTL* (Sheldrick, 2008 ▶); software used to prepare material for publication: *SHELXTL*.

## Supplementary Material

Crystal structure: contains datablocks global, I. DOI: 10.1107/S1600536810024645/sj5026sup1.cif

Structure factors: contains datablocks I. DOI: 10.1107/S1600536810024645/sj5026Isup2.hkl

Additional supplementary materials:  crystallographic information; 3D view; checkCIF report

## Figures and Tables

**Table 1:** Hydrogen-bond geometry (Å, °) *Cg*1 is the centroid of the N1/C7/C1/C2/N2/C8 ring.

*D*—H⋯*A*	*D*—H	H⋯*A*	*D*⋯*A*	*D*—H⋯*A*
N3—H3*B*⋯N2^i^	0.86 (1)	2.40 (2)	3.150 (3)	145 (3)
N3—H3*A*⋯O1^ii^	0.86 (1)	2.46 (2)	3.147 (3)	137 (3)
C15—H15⋯F2	0.93	2.40	2.93 (3)	116
C12—H12⋯*Cg*1^i^	0.93	2.88	3.560 (3)	131

Symmetry codes: (i) 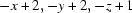; (ii) 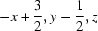.

**Fig. 1.**
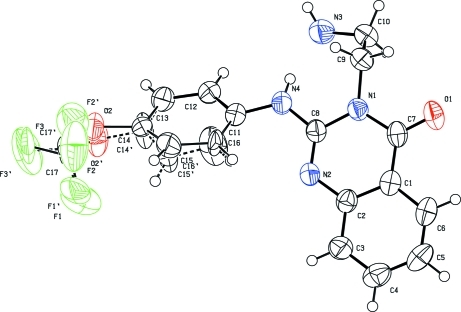
View of the molecular structure of (I), showing the atom labelling schemeand with displacement ellipsoids drawn at the 50% probability level. Both disorder components are shown with bonds involving the minor disorder component drawn as dashed lines.

**Fig. 2.**
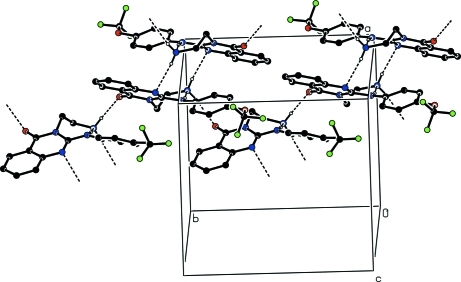
A partial view of the crystal packing of (I), showing the formation of N—H···N and N—H···O hydrogen-bonds as dashed lines.

## supplementary crystallographic information

### Comment

Quinazoline-4(3*H*)-one derivatives have numerous biological properties. Some of these activities include antimicrobial (Pandeya *et al.*, 1999 and Shiba *et al.*, 1997), antidiabetic (Malamas & Millen, 1991), anticonvulsant (Mannschreck *et al.*, 1984), antibacterial (Kung *et al.*, 1999), antifungal (Bartroli *et al.*, 1998), protein tyrosine kinase inhibitors (Palmer *et al.*, 1997), EGFR inhibitors (Tsou *et al.*,2001) and PDGFR phosphorylation inhibitors (Matsuno *et al.*, 2002). We have recently focused on the synthesis of heterocyclic compounds using an aza-Wittig reaction. We have reported the synthesis of the title compound (Yang *et al.*, 2008). We present here the crystal structure of the title compound, (I) (Fig. 1), which can be used as a precursor for obtaining bioactive molecules.

In the crystal structure, the fused benzene ring and the pyrimidinone ring are not completely co-planar, but are inclined at 0.67 (1) °. Significant and intermolecular N—H···O and N—H···N hydrogen bonds contribute strongly to the stability of the structure (Fig. 2). An intramolecular C—H···F hydrogen bond is also found. (Table 1). The crystal structure (Fig. 2) is also stabilized by weak intermolecular C—H···π hydrogen bonds (Table 1) and π— π stacking interactions with centroid-centroid separations of 3.673 (6), 3.779 (8), 3.674 (6) and 3.780 (8) Å for *Cg*1···*Cg*3^i^, *Cg*1···*Cg*4^i^, *Cg*3···*Cg*1^ii^ and *Cg*4···*Cg*1^ii^, respectively, where *Cg*1, *Cg*3 and *Cg*4 are the centroids of the N1/C7/C1—C2/N2/C8, C11—C16 and C11—C13/C14'-C16' rings, respectively [symmetry code: (i) 3/2-*X*, 1/2+Y, *Z*, (ii) 3/2-*X*, -1/2+Y, *Z*,].

### Experimental

The title compound was prepared by a literature method (Yang *et al.*, 2008). Single crystals suitable for X-ray diffraction were obtained from a methanol-dichloromethane (1:1 *v*/*v*) solution at room temperature.

### Refinement

H atoms bonded to C were placed in calculated positions, with C—H distances of 0.97 and 0.93Å for H atoms bonded to *sp*^3^ and *sp*^2^ C atoms, respectively. They were refined using a riding model, with *U*_iso_(H) = 1.2*U*_eq_(C), or 1.5*U*_eq_(methyl C). The H atoms bound to N were refined with distance restraints N—H = 0.86 (2)Å and with *U*_iso_(H) = 1.2*U*_eq_ (N). The C14···C16 atoms of the trifluoromethoxy-substituted benzene ring and all atoms of the trifluoromethoxy substituent were disordered over two sites. The site occupancies refined to 0.51 and 0.49 and were fixed at these values in the final refinement cycles.

### Crystal data

**Table:**

C_17_H_15_F_3_N_4_O_2_	*F*(000) = 1504
*M**_r_* = 364.33	*D*_x_ = 1.467 Mg m^−^^3^
Orthorhombic, *P**b**c**n*	Mo *K*α radiation, λ = 0.71073 Å
Hall symbol: -P 2n 2ab	Cell parameters from 4600 reflections
*a* = 11.9675 (13) Å	θ = 2.5–23.5°
*b* = 12.9579 (13) Å	µ = 0.12 mm^−^^1^
*c* = 21.280 (2) Å	*T* = 298 K
*V* = 3300.0 (6) Å^3^	Block, colorless
*Z* = 8	0.23 × 0.15 × 0.11 mm

### Data collection

**Table:**

Bruker SMART APEX CCD area-detector diffractometer	3076 independent reflections
Radiation source: fine-focus sealed tube	2573 reflections with *I* > 2σ(*I*)
graphite	*R*_int_ = 0.083
φ and ω scans	θ_max_ = 25.5°, θ_min_ = 2.3°
Absorption correction: multi-scan (*SADABS*; Sheldrick, 2001)	*h* = −10→14
*T*_min_ = 0.973, *T*_max_ = 0.987	*k* = −15→14
15599 measured reflections	*l* = −25→25

### Refinement

**Table:**

Refinement on *F*^2^	Primary atom site location: structure-invariant direct methods
Least-squares matrix: full	Secondary atom site location: difference Fourier map
*R*[*F*^2^ > 2σ(*F*^2^)] = 0.061	Hydrogen site location: inferred from neighbouring sites
*wR*(*F*^2^) = 0.164	H atoms treated by a mixture of independent and constrained refinement
*S* = 1.12	*w* = 1/[σ^2^(*F*_o_^2^) + (0.0809*P*)^2^ + 0.8235*P*] where *P* = (*F*_o_^2^ + 2*F*_c_^2^)/3
3076 reflections	(Δ/σ)_max_ < 0.001
311 parameters	Δρ_max_ = 0.39 e Å^−^^3^
19 restraints	Δρ_min_ = −0.29 e Å^−^^3^

### Special details

**Table:**

Geometry. All e.s.d.'s (except the e.s.d. in the dihedral angle between two l.s. planes) are estimated using the full covariance matrix. The cell e.s.d.'s are taken into account individually in the estimation of e.s.d.'s in distances, angles and torsion angles; correlations between e.s.d.'s in cell parameters are only used when they are defined by crystal symmetry. An approximate (isotropic) treatment of cell e.s.d.'s is used for estimating e.s.d.'s involving l.s. planes.
Refinement. Refinement of *F*^2^ against ALL reflections. The weighted *R*-factor *wR* and goodness of fit *S* are based on *F*^2^, conventional *R*-factors *R* are based on *F*, with *F* set to zero for negative *F*^2^. The threshold expression of *F*^2^ > σ(*F*^2^) is used only for calculating *R*-factors(gt) *etc*. and is not relevant to the choice of reflections for refinement. *R*-factors based on *F*^2^ are statistically about twice as large as those based on *F*, and *R*- factors based on ALL data will be even larger.

### Fractional atomic coordinates and isotropic or equivalent isotropic displacement parameters (Å^2^)

**Table:**

	*x*	*y*	*z*	*U*_iso_*/*U*_eq_	Occ. (<1)
C1	0.9187 (2)	1.31297 (18)	0.55231 (11)	0.0445 (6)	
C2	0.9408 (2)	1.24194 (18)	0.60015 (11)	0.0443 (6)	
C3	0.9930 (2)	1.2767 (2)	0.65508 (12)	0.0556 (7)	
H3	1.0093	1.2304	0.6871	0.067*	
C4	1.0200 (2)	1.3792 (2)	0.66172 (14)	0.0623 (7)	
H4	1.0546	1.4014	0.6984	0.075*	
C5	0.9967 (2)	1.4498 (2)	0.61481 (14)	0.0631 (8)	
H5	1.0156	1.5189	0.6199	0.076*	
C6	0.9457 (2)	1.41721 (19)	0.56101 (14)	0.0549 (7)	
H6	0.9286	1.4648	0.5297	0.066*	
C7	0.8649 (2)	1.27789 (18)	0.49482 (11)	0.0453 (6)	
C8	0.8673 (2)	1.10775 (18)	0.54329 (10)	0.0425 (6)	
C9	0.7649 (2)	1.1385 (2)	0.44173 (12)	0.0522 (6)	
H9A	0.7257	1.1979	0.4249	0.063*	
H9B	0.7092	1.0915	0.4585	0.063*	
C10	0.8258 (2)	1.0850 (2)	0.38838 (11)	0.0555 (7)	
H10A	0.7795	1.0861	0.3510	0.067*	
H10B	0.8943	1.1220	0.3791	0.067*	
C11	0.8519 (2)	0.92710 (18)	0.57795 (12)	0.0463 (6)	
C12	0.8785 (2)	0.82950 (18)	0.55666 (12)	0.0463 (6)	
H12	0.8970	0.8200	0.5146	0.056*	
C13	0.8781 (2)	0.74562 (19)	0.59680 (13)	0.0523 (7)	
H13	0.8987	0.6808	0.5821	0.063*	
C14	0.8473 (13)	0.7581 (8)	0.6583 (5)	0.065 (5)	0.51
C15	0.812 (2)	0.855 (2)	0.6764 (13)	0.071 (6)	0.51
H15	0.7805	0.8616	0.7161	0.085*	0.51
C16	0.8208 (17)	0.9417 (9)	0.6397 (8)	0.050 (3)	0.51
H16	0.8065	1.0072	0.6558	0.060*	0.51
C17	0.8238 (9)	0.6670 (8)	0.7538 (5)	0.0637 (12)	0.51
O2	0.8387 (12)	0.6644 (9)	0.6922 (5)	0.089 (4)	0.51
F1	0.8935 (8)	0.7216 (9)	0.7869 (6)	0.119 (4)	0.51
F2	0.7234 (6)	0.7043 (7)	0.7658 (5)	0.122 (4)	0.51
F3	0.8159 (10)	0.5681 (5)	0.7691 (4)	0.082 (2)	0.51
C14'	0.8582 (12)	0.7631 (9)	0.6591 (6)	0.063 (5)	0.49
C15'	0.842 (3)	0.8590 (19)	0.6855 (14)	0.071 (6)	0.49
H15'	0.8374	0.8700	0.7286	0.085*	0.49
C16'	0.834 (3)	0.9368 (16)	0.6418 (13)	0.101 (8)	0.49
H16'	0.8148	1.0020	0.6566	0.121*	0.49
C17'	0.8114 (10)	0.6564 (9)	0.7475 (6)	0.0637 (12)	0.49
O2'	0.8747 (11)	0.6749 (8)	0.6976 (5)	0.070 (3)	0.49
F1'	0.8435 (10)	0.7198 (6)	0.7928 (4)	0.105 (4)	0.49
F2'	0.7057 (9)	0.6717 (8)	0.7373 (5)	0.122 (4)	0.49
F3'	0.8383 (15)	0.5654 (9)	0.7708 (7)	0.145 (6)	0.49
N1	0.83729 (16)	1.17338 (14)	0.49385 (9)	0.0428 (5)	
N2	0.91631 (18)	1.13823 (14)	0.59417 (9)	0.0469 (5)	
N3	0.8525 (2)	0.97796 (19)	0.40455 (11)	0.0605 (6)	
H3A	0.7992 (19)	0.936 (2)	0.3949 (14)	0.073*	
H3B	0.9115 (17)	0.952 (2)	0.3871 (14)	0.073*	
N4	0.8435 (2)	1.00667 (16)	0.53333 (9)	0.0510 (6)	
H4A	0.843 (2)	0.988 (2)	0.4945 (6)	0.061*	
O1	0.84285 (17)	1.33377 (14)	0.45034 (9)	0.0620 (6)	

### Atomic displacement parameters (Å^2^)

**Table:**

	*U*^11^	*U*^22^	*U*^33^	*U*^12^	*U*^13^	*U*^23^
C1	0.0419 (13)	0.0403 (12)	0.0513 (13)	0.0033 (10)	0.0089 (11)	0.0027 (10)
C2	0.0470 (13)	0.0410 (13)	0.0449 (13)	−0.0008 (10)	0.0068 (10)	0.0003 (10)
C3	0.0640 (17)	0.0577 (16)	0.0452 (13)	−0.0084 (13)	0.0010 (12)	−0.0015 (11)
C4	0.0643 (18)	0.0622 (18)	0.0604 (16)	−0.0108 (14)	0.0069 (14)	−0.0154 (14)
C5	0.0644 (18)	0.0428 (14)	0.082 (2)	−0.0098 (12)	0.0113 (16)	−0.0141 (13)
C6	0.0550 (15)	0.0397 (13)	0.0702 (17)	0.0013 (11)	0.0113 (13)	0.0066 (12)
C7	0.0455 (13)	0.0415 (13)	0.0490 (13)	0.0078 (10)	0.0076 (11)	0.0077 (10)
C8	0.0465 (13)	0.0402 (12)	0.0410 (12)	0.0001 (10)	0.0031 (10)	0.0038 (10)
C9	0.0521 (15)	0.0527 (15)	0.0519 (13)	0.0026 (11)	−0.0113 (12)	0.0054 (11)
C10	0.0634 (16)	0.0601 (16)	0.0431 (13)	−0.0049 (13)	−0.0077 (12)	0.0038 (12)
C11	0.0535 (14)	0.0399 (13)	0.0455 (13)	−0.0049 (10)	−0.0006 (11)	0.0033 (10)
C12	0.0491 (14)	0.0431 (13)	0.0466 (13)	−0.0014 (10)	−0.0007 (11)	−0.0015 (10)
C13	0.0601 (16)	0.0386 (13)	0.0583 (16)	0.0076 (11)	−0.0039 (13)	−0.0029 (11)
C14	0.121 (10)	0.032 (7)	0.043 (7)	−0.017 (6)	−0.026 (6)	−0.002 (4)
C15	0.118 (13)	0.063 (7)	0.033 (7)	−0.014 (7)	0.003 (6)	−0.004 (4)
C16	0.097 (7)	0.014 (4)	0.040 (6)	−0.001 (4)	0.011 (5)	0.001 (4)
C17	0.084 (3)	0.054 (2)	0.053 (2)	0.002 (2)	−0.004 (2)	0.0146 (19)
O2	0.167 (12)	0.046 (3)	0.055 (3)	−0.033 (5)	−0.014 (5)	0.013 (2)
F1	0.111 (6)	0.144 (6)	0.101 (6)	0.025 (4)	−0.036 (4)	−0.008 (4)
F2	0.084 (5)	0.107 (6)	0.177 (9)	0.021 (4)	0.060 (6)	0.059 (6)
F3	0.132 (5)	0.045 (3)	0.070 (4)	0.001 (3)	0.018 (3)	0.038 (3)
C14'	0.071 (6)	0.050 (9)	0.069 (9)	0.024 (5)	0.032 (6)	0.027 (6)
C15'	0.139 (17)	0.036 (6)	0.037 (8)	0.003 (8)	0.006 (9)	0.006 (5)
C16'	0.164 (19)	0.075 (11)	0.064 (12)	0.026 (9)	0.035 (11)	0.001 (8)
C17'	0.084 (3)	0.054 (2)	0.053 (2)	0.002 (2)	−0.004 (2)	0.0146 (19)
O2'	0.098 (6)	0.038 (4)	0.076 (5)	0.016 (4)	0.022 (5)	0.011 (3)
F1'	0.197 (12)	0.074 (4)	0.045 (3)	0.000 (5)	−0.031 (5)	−0.014 (2)
F2'	0.107 (5)	0.116 (7)	0.144 (7)	−0.005 (4)	−0.023 (5)	0.038 (5)
F3'	0.186 (11)	0.112 (8)	0.137 (9)	0.059 (6)	−0.005 (7)	0.054 (6)
N1	0.0465 (11)	0.0404 (11)	0.0416 (10)	0.0029 (8)	−0.0018 (8)	0.0038 (8)
N2	0.0591 (13)	0.0395 (11)	0.0421 (11)	−0.0024 (9)	−0.0030 (9)	0.0057 (8)
N3	0.0730 (17)	0.0565 (15)	0.0518 (13)	0.0004 (12)	−0.0036 (12)	−0.0049 (11)
N4	0.0754 (15)	0.0385 (11)	0.0389 (11)	−0.0060 (10)	−0.0015 (10)	0.0022 (9)
O1	0.0760 (13)	0.0527 (11)	0.0573 (11)	0.0087 (9)	−0.0023 (9)	0.0205 (9)

### Geometric parameters (Å, °)

**Table:**

C1—C2	1.398 (3)	C11—N4	1.405 (3)
C1—C6	1.401 (3)	C12—C13	1.382 (3)
C1—C7	1.455 (4)	C12—H12	0.9300
C2—N2	1.381 (3)	C13—C14'	1.366 (14)
C2—C3	1.399 (3)	C13—C14	1.369 (13)
C3—C4	1.374 (4)	C13—H13	0.9300
C3—H3	0.9300	C14—C15	1.38 (3)
C4—C5	1.382 (4)	C14—O2	1.416 (9)
C4—H4	0.9300	C15—C16	1.38 (4)
C5—C6	1.364 (4)	C15—H15	0.9300
C5—H5	0.9300	C16—H16	0.9300
C6—H6	0.9300	C17—F1	1.302 (9)
C7—O1	1.221 (3)	C17—F2	1.321 (9)
C7—N1	1.394 (3)	C17—O2	1.324 (9)
C8—N2	1.293 (3)	C17—F3	1.326 (8)
C8—N4	1.357 (3)	C14'—C15'	1.38 (3)
C8—N1	1.400 (3)	C14'—O2'	1.421 (8)
C9—N1	1.478 (3)	C15'—C16'	1.37 (4)
C9—C10	1.517 (4)	C15'—H15'	0.9300
C9—H9A	0.9700	C16'—H16'	0.9300
C9—H9B	0.9700	C17'—F2'	1.299 (9)
C10—N3	1.464 (4)	C17'—F3'	1.320 (9)
C10—H10A	0.9700	C17'—F1'	1.325 (9)
C10—H10B	0.9700	C17'—O2'	1.325 (9)
C11—C16	1.379 (18)	N3—H3A	0.862 (11)
C11—C12	1.381 (3)	N3—H3B	0.864 (11)
C11—C16'	1.38 (3)	N4—H4A	0.863 (11)
			
C2—C1—C6	119.7 (2)	C14—C13—C12	119.9 (5)
C2—C1—C7	119.4 (2)	C14'—C13—H13	121.6
C6—C1—C7	120.9 (2)	C14—C13—H13	120.1
N2—C2—C1	122.2 (2)	C12—C13—H13	120.1
N2—C2—C3	119.0 (2)	C13—C14—C15	117.3 (15)
C1—C2—C3	118.7 (2)	C13—C14—O2	113.9 (9)
C4—C3—C2	120.1 (3)	C15—C14—O2	127.9 (17)
C4—C3—H3	119.9	C14—C15—C16	124 (2)
C2—C3—H3	119.9	C14—C15—H15	117.9
C3—C4—C5	121.2 (3)	C16—C15—H15	117.9
C3—C4—H4	119.4	C15—C16—C11	116.7 (16)
C5—C4—H4	119.4	C15—C16—H16	121.7
C6—C5—C4	119.4 (2)	C11—C16—H16	121.7
C6—C5—H5	120.3	F1—C17—F2	106.2 (9)
C4—C5—H5	120.3	F1—C17—O2	117.7 (11)
C5—C6—C1	120.8 (3)	F2—C17—O2	108.9 (11)
C5—C6—H6	119.6	F1—C17—F3	116.0 (10)
C1—C6—H6	119.6	F2—C17—F3	104.0 (9)
O1—C7—N1	120.9 (2)	O2—C17—F3	103.2 (9)
O1—C7—C1	124.2 (2)	C17—O2—C14	119.5 (11)
N1—C7—C1	114.88 (19)	C13—C14'—C15'	124.8 (15)
N2—C8—N4	121.4 (2)	C13—C14'—O2'	113.7 (10)
N2—C8—N1	124.1 (2)	C15'—C14'—O2'	120.6 (16)
N4—C8—N1	114.5 (2)	C16'—C15'—C14'	113 (2)
N1—C9—C10	114.8 (2)	C16'—C15'—H15'	123.3
N1—C9—H9A	108.6	C14'—C15'—H15'	123.3
C10—C9—H9A	108.6	C15'—C16'—C11	126 (2)
N1—C9—H9B	108.6	C15'—C16'—H16'	117.0
C10—C9—H9B	108.6	C11—C16'—H16'	117.0
H9A—C9—H9B	107.5	F2'—C17'—F3'	115.9 (11)
N3—C10—C9	111.2 (2)	F2'—C17'—F1'	108.1 (9)
N3—C10—H10A	109.4	F3'—C17'—F1'	102.1 (10)
C9—C10—H10A	109.4	F2'—C17'—O2'	113.3 (11)
N3—C10—H10B	109.4	F3'—C17'—O2'	108.8 (11)
C9—C10—H10B	109.4	F1'—C17'—O2'	107.8 (10)
H10A—C10—H10B	108.0	C17'—O2'—C14'	121.8 (11)
C16—C11—C12	120.0 (6)	C7—N1—C8	121.2 (2)
C16—C11—C16'	7(2)	C7—N1—C9	116.56 (19)
C12—C11—C16'	116.2 (10)	C8—N1—C9	121.9 (2)
C16—C11—N4	121.6 (6)	C8—N2—C2	118.07 (19)
C12—C11—N4	117.8 (2)	C10—N3—H3A	112 (2)
C16'—C11—N4	125.9 (10)	C10—N3—H3B	116 (2)
C11—C12—C13	121.1 (2)	H3A—N3—H3B	105 (3)
C11—C12—H12	119.4	C8—N4—C11	126.0 (2)
C13—C12—H12	119.4	C8—N4—H4A	115.3 (19)
C14'—C13—C14	6.1 (12)	C11—N4—H4A	115.9 (19)
C14'—C13—C12	118.0 (5)		
			
C6—C1—C2—N2	−179.8 (2)	C15—C14—O2—C17	−20 (2)
C7—C1—C2—N2	1.9 (3)	C14—C13—C14'—C15'	−112 (10)
C6—C1—C2—C3	−2.0 (3)	C12—C13—C14'—C15'	−2(2)
C7—C1—C2—C3	179.8 (2)	C14—C13—C14'—O2'	79 (9)
N2—C2—C3—C4	178.9 (2)	C12—C13—C14'—O2'	−171.7 (9)
C1—C2—C3—C4	1.0 (4)	C13—C14'—C15'—C16'	8(3)
C2—C3—C4—C5	0.0 (4)	O2'—C14'—C15'—C16'	176 (2)
C3—C4—C5—C6	0.2 (4)	C14'—C15'—C16'—C11	−7(4)
C4—C5—C6—C1	−1.2 (4)	C16—C11—C16'—C15'	124 (12)
C2—C1—C6—C5	2.2 (4)	C12—C11—C16'—C15'	1(3)
C7—C1—C6—C5	−179.6 (2)	N4—C11—C16'—C15'	180 (2)
C2—C1—C7—O1	−179.5 (2)	F2'—C17'—O2'—C14'	43.8 (16)
C6—C1—C7—O1	2.3 (4)	F3'—C17'—O2'—C14'	174.2 (14)
C2—C1—C7—N1	1.6 (3)	F1'—C17'—O2'—C14'	−75.8 (15)
C6—C1—C7—N1	−176.6 (2)	C13—C14'—O2'—C17'	−144.6 (11)
N1—C9—C10—N3	78.7 (3)	C15'—C14'—O2'—C17'	45 (2)
C16—C11—C12—C13	−2.0 (10)	O1—C7—N1—C8	176.8 (2)
C16'—C11—C12—C13	5.1 (15)	C1—C7—N1—C8	−4.3 (3)
N4—C11—C12—C13	−173.9 (2)	O1—C7—N1—C9	−9.6 (3)
C11—C12—C13—C14'	−4.5 (8)	C1—C7—N1—C9	169.3 (2)
C11—C12—C13—C14	2.2 (8)	N2—C8—N1—C7	3.8 (4)
C14'—C13—C14—C15	77 (9)	N4—C8—N1—C7	−174.5 (2)
C12—C13—C14—C15	3.3 (19)	N2—C8—N1—C9	−169.5 (2)
C14'—C13—C14—O2	−113 (9)	N4—C8—N1—C9	12.2 (3)
C12—C13—C14—O2	173.4 (8)	C10—C9—N1—C7	100.9 (3)
C13—C14—C15—C16	−10 (3)	C10—C9—N1—C8	−85.5 (3)
O2—C14—C15—C16	−178.0 (19)	N4—C8—N2—C2	178.1 (2)
C14—C15—C16—C11	10 (3)	N1—C8—N2—C2	−0.1 (4)
C12—C11—C16—C15	−4(2)	C1—C2—N2—C8	−2.7 (3)
C16'—C11—C16—C15	−64 (10)	C3—C2—N2—C8	179.4 (2)
N4—C11—C16—C15	168.0 (16)	N2—C8—N4—C11	10.1 (4)
F1—C17—O2—C14	−52.8 (17)	N1—C8—N4—C11	−171.5 (2)
F2—C17—O2—C14	68.0 (15)	C16—C11—N4—C8	39.3 (10)
F3—C17—O2—C14	178.0 (13)	C12—C11—N4—C8	−148.9 (2)
C13—C14—O2—C17	171.3 (11)	C16'—C11—N4—C8	32.2 (16)

### Hydrogen-bond geometry (Å, °)

**Table:**

Cg1 is the centroid of the N1/C7/C1/C2/N2/C8 ring.

**Table:**

*D*—H···*A*	*D*—H	H···*A*	*D*···*A*	*D*—H···*A*
N3—H3B···N2^i^	0.86 (1)	2.40 (2)	3.150 (3)	145 (3)
N3—H3A···O1^ii^	0.86 (1)	2.46 (2)	3.147 (3)	137 (3)
C15—H15···F2	0.93	2.40	2.93 (3)	116
C12—H12···Cg1^i^	0.93	2.88	3.560 (3)	131

Symmetry codes: (i) −*x*+2, −*y*+2, −*z*+1; (ii) −*x*+3/2, *y*−1/2, *z*.
